# Comprehensive phytochemical profiles and antioxidant activity of Korean local cultivars of red chili pepper (*Capsicum annuum L.*)

**DOI:** 10.3389/fpls.2024.1333035

**Published:** 2024-01-22

**Authors:** Hyemi Jang, Mira Choi, Kyoung-Soon Jang

**Affiliations:** ^1^ Bio-Chemical Analysis Team, Korea Basic Science Institute, Cheongju, Republic of Korea; ^2^ Division of Bio-Analytical Science, University of Science and Technology, Daejeon, Republic of Korea

**Keywords:** red chili pepper, Capsicum annuum, Korean local cultivar, phytochemical, ion mobility Q-TOF, capsaicinoid, antioxidant activity

## Abstract

Red chili pepper (*Capsicum annuum L.*), which belongs to the Solanaceae family, contains a variety of phytochemicals with health-promoting properties including capsaicinoids, phenolics and fatty acids. Red chili pepper is one of the most consumed vegetables in Korea and occupies the largest cultivated area among spices. In this study, the ethanolic extracts from two Korean local cultivars, namely Subicho and Eumseong, were analyzed using a hybrid trapped ion mobility Q-TOF mass spectrometer equipped with a UPLC system, and their phytochemical profiles were then compared with those of a common phytophthora disease-resistant cultivar called Dokbulwang, which is extensively used for red chili pepper powder in public spaces across Korea. Utilizing high-resolution ion-mobility Q-TOF MS analysis, 458 and 192 compounds were identified from the three different red chili peppers in positive and negative ion modes, respectively, by matching with a reference spectral library. Principal component analysis revealed clear distinctions among the three cultivars, allowing us to identify key phytochemical components responsible for discriminating the local cultivars from the public cultivar. Furthermore, the assessment of total flavonoid, phenolic, and antioxidant activity in the red pepper extracts, highlighted their diverse molecular and chemical profiles. Despite the higher total flavonoid and phenolic content values observed in the public cultivar, the radical scavenging rate was higher in the local cultivars, particularly in Subicho. This suggest the presence of stronger antioxidant compounds in the local cultivar, indicating their potential health benefits due to their rich content of bioactive compounds. Notably, the local cultivars exhibited significantly higher proportions of organic compounds (more than four times) and terpenoids (more than two times) compared to the public cultivar. Specifically, higher levels of five major capsaicinoid compounds were found in the local cultivars when compared to the public cultivar. The observed disparities in phytochemical composition and antioxidant activities indicate the molecular diversity present among these cultivars. Further exploration of the bioactive compounds in these local cultivars could prove invaluable for the development of native crops, potentially leading to the discovery of novel sources of bioactive molecules for various applications in health and agriculture.

## 1 Introduction

Red chili pepper (*Capsicum annuum L.*), which belongs to the plant family Solanaceae, is known to have originated in Central or South America and is estimated to have been introduced to Korea in the early 17^th^ century (Pickersgill, 1971; Chiou and Hastorf, 2014). Red chilies are the most widely cultivated seasoning and traditional medicinal crop in many tropical and subtropical countries (Bravo, 1998; Antonio et al., 2018; Zhang et al., 2021). They are primarily used as spices to impart spiciness to dishes and are recognized for their ability to harmoniously blend the inherent piquancy and sweetness, resulting in a unique umami flavor. Red chili pepper is known to contain a variety of bioactive compounds with health-promoting properties such as carotenoids, capsaicinoids, phenolics (flavonoids), vitamin C, vitamin E and fatty acids (Wahyuni et al., 2013; Azlan et al., 2022), and recent interest has surged in the functionality and applications of various physiological bioactive compounds found in red pepper, including capsaicin, which, aside from enhancing the flavor of food and stimulating appetite, also contribute to the physiological effects of spiciness (Huang et al., 2014).

Numerous indigenous species have acclimated to the distinct climates of various regions over extended periods, while several cultivated varieties have undergone enhancements through selective breeding by specialized seedling companies (Lanteri et al., 2003; Bozokalfa et al., 2009). Particularly, these varieties are bred to withstand specific environmental conditions or resist certain pests, enabling more effective cultivation. These traits play a vital role in elevating agricultural productivity and delivering consumers with peppers of superior quality. Nevertheless, such cultivars developed for disease resistance often prioritize productivity and spiciness, especially for commercial red pepper powder production, leading to the loss of their distinctive flavors and tastes (Simon and Tesfaye, 2014; Gebrtsadkan et al., 2018).

The per capita consumption of red chili pepper among Koreans ranks as the highest globally, and a diverse array of local red chili pepper cultivars is available, and efforts by local governments have been underway to revive these cultivars (Hwang et al., 2014; Jo et al., 2014). One such cultivar, Subicho, originated from the peppers cultivated in Subi-myeon, Yeongyang-gun, Gyeongsangbuk-do, Korea, and gained popularity in the 1970s. Subicho is renowned for its intense spiciness, high sweetness, and crisp texture (Jang et al., 2015). Another cultivar, Eumseong, hails from Eumseong-gun, Chungcheongbuk-do and is also known to have balanced pungency and color. Despite their lower fruit productivity, these local cultivars are well-suited to the Korean terrain and are garnering attention due to their unique flavors and tastes. While red chili peppers are known to harbor diverse bioactive compounds, there is limited information available regarding the specific phytochemical profiles and antioxidant activity of these Korean local cultivars. Therefore, exploring the phytochemicals of these local cultivars is imperative. Such research is essential not only for distinguishing between domestic and imported varieties but also for showcasing the superiority of cultivars that rank high in domestic pepper cultivation.

The quality of red pepper cultivars with different cultivation years and regions were characterized by analyzing the capsaicinoid and free sugar contents and ASTA (American Spice Trade Association) color value (Hwang et al., 2014). Although their findings indicated that variations in capsaicinoid contents were linked to specific cultivars and the free sugar contents and ASTA values were influenced by environmental factors such as the cultivation region, to fully harness the potential of domestically indigenous peppers for diverse applications, it is crucial to comprehensively analyze their active ingredients and enhance their utility value.

Various methodologies have been applied in the phytochemical profiling of red chili peppers. Morales-Soriano and coworkers utilized an integrated approach, encompassing fingerprinting, profiling, and chemometrics, to characterize chili peppers effectively (Morales-Soriano et al., 2018). Additionally, Altemimi and coworkers provided a concise overview of a diverse range of assays employed for the extraction, measurement, and identification of bioactive compounds in fruits and vegetables (Altemimi et al., 2017). Furthermore, Stoica and colleagues reviewed the potential of GC-MS method for both qualitative and quantitative analysis of major capsaicinoids in ground chilies, chili sauces, and even potato chips (Stoica et al., 2016). Consequently, precise analysis of physiological bioactive compounds within traditional plant varieties is indispensable. Not only does it elucidate the functional efficacy of pepper-derived constituents, but it also contributes significantly to international standardization efforts, especially in developing analytical methods for the standardization of functional ingredients in local plants.

A comprehensive analysis of the bioactive components present in functional crops like red chili peppers serves not merely as a means of comprehending the physiological functions of these agricultural products or as a tool for quality standardization. Considerable attention has been directed towards the development of bioactive constituents from these functional crops for their potential use in oral administration of natural radical scavengers as food supplements. Examples include extracts from green tea (Buetler et al., 2002) and preservative food additives derived from aromatic plants, such as rosemary and salvia extracts (Karpinska et al., 2000; Zupko et al., 2001). In recent years, there has been a surge in rigorous research investigating the commercial utilization of radical scavengers and flavonoids as beneficial anti-aging and photoprotective ingredients in cosmetics (Katiyar and Elmets, 2001; Lupo, 2001). Furthermore, specific bioactive compounds found in red chili peppers will hold potential utility as novel agents for pest control (Cantrell et al., 2012; Gerwick and Sparks, 2014; Khursheed et al., 2022) and drug discovery (Atanasov et al., 2021).

In this study, a comprehensive analysis of the phytochemical profiles of two indigenous Korean red chili pepper cultivars (referred to as the local cultivars) were conducted and compared with those of Dokbulwang cultivar (the public cultivar), a common phytophthora disease-resistance cultivar. Notably, the Dokbulwang cultivar is commonly used in Korea for the production of red chili pepper in public domains. The detailed examination of phytochemical profiles and their corresponding antioxidant capacities facilitated a deeper understanding of their distinct physiochemical attributes. These findings have the potential to enhance our comprehension of the health advantages associated with red pepper consumption and offer valuable insights for the food and pharmaceutical industries, offering avenues for the development of functional foods, dietary supplements or medicinal drugs.

## 2 Materials and methods

### 2.1 Preparation for ethanolic extracts of red chili peppers

Three red chili samples used in this study: two indigenous Korean local cultivars, namely Subicho and Eumseong, and a common phytophthora disease-resistance cultivar, Dokbulwang. These peppers were cultivated at an altitude of 450 m in Yeongwol-gun, Gangwon-do (37.05°N, 128.32°E). Harvesting took place in October 2021, followed by natural drying and stored at –20°C until further use. The red chili pepper samples were lyophilized (freeze-dried) using a lyophilizer and then homogenized using an analytical mill (A11, IKA, Staufen, Germany). The homogenized red pepper samples underwent extraction (1 g sample in 10 mL 80% ethanol) in an ultrasonic bath (Power sonic, Hwashin Instrument, Seoul, Korea) at room temperature for 30 min. To extract phytochemical compounds, including health-promoting antioxidants, from plant materials, the selection of an appropriate solvent is crucial because the extraction yield and the chemical properties of the plant depend on the solvent chosen and the characteristics of the target compounds (Sultana et al., 2009; El Mannoubi, 2023). While various solvents such as absolute methanol, absolute ethanol, aqueous methanol (80% methanol, v/v), or aqueous ethanol (80% ethanol, v/v) can be employed to extract phytochemical constituents from plant samples, ethanol is more commonly utilized as an organic solvent than methanol, serving as a reaction medium for the extraction of natural products and for equipment cleaning purposes in the pharmaceutical and food industries. In this study, 80% ethanol was employed for extraction of both polar and semi-polar components in red chili peppers. Subsequent to extraction, the supernatants were transferred and filtered using a Millipore Millex-FH cartridge filter (Hydrophobic PTEE 0.45 μm) to eliminate insoluble debris. The resulting solutions were placed in a refrigerator for a day to precipitate cellulosic components that might interfere with chromatographic separation. Following centrifugation (12,000 rpm, 15 min, 4°C), the soluble fractions were dried using a centrifugal vacuum concentrator and stored at –20°C for further analysis.

### 2.2 Determination of total phenolic and flavonoid contents

The total phenolic and flavonoid contents of ethanolic extracts from red chili pepper were determined by using a Folin-Ciocalteu assay (Ainsworth and Gillespie, 2007) and a NaNO_2_-Al(NO_3_)_3_-NaOH colorimetric assay (Fattahi et al., 2014), respectively, with minor modification. For the total phenolic content (TPC) assay, 100 μL of the extracts were combined with 650 μL of deionized water, then the Folin-Ciocalteu (F-C) reagent (50 μL) and 7.5% sodium carbonate solution (200 μL) were added to the mixture. The F-C assay is based on the electron transfer in alkaline environment, wherein phenolic compounds serve as electron donors, catalyzing the reduction of phosphomolybdic/phosphotungstic acid complexes and culminating in the generation of blue-colored complexes. After 30 min of incubation in darkness at room temperature, the absorbance was measured at 765 nm using a spectrophotometer. TPC results were expressed as mg equivalent of gallic acid (GAE) per gram of dry sample weight (Ainsworth and Gillespie, 2007). In the total flavonoid content (TFC) assay, 200 μL of the extracts were mixed with 540 μL of deionized water, followed by the addition of 30 μL of 5% sodium nitrate. The mixture was allowed to stand for 5 min to ensure oxidation of hydroxyl residues of flavonoids. After adding 30 μL of 10% aluminum chloride, the mixtures were incubated for 6 min. Here, Al(III) is utilized as a complexing agent forming chelates of Al(III)-flavonoids (Shraim et al., 2021). To quench the reaction, 200 μL of 1M sodium hydroxide solution was added, followed by further incubation at 25°C for 30 min. The absorbance of the resulting solution was measured at 510 nm. TFC values were expressed as mg catechin equivalents (CE) per gram of dried sample powder (Fattahi et al., 2014). The absorbance of samples, subtracted by the absorbance of the blank, was used to calculate both TPC and TFC. All assays were performed in triplicate.

### 2.3 Anti-oxidant scavenging assay

The anti-oxidant potential of the extract was assessed using the [2,2-di(4-tert-octylphenyl)-1-picrylhydrazyl] (DPPH) radical scavenging assay, following established protocols (Marinova and Batchvarov, 2011). Specifically, 100 μL of the sample was combined with freshly prepared DPPH solution (0.1 mM in methanol) and deionized water. The mixture was then incubated for 15 min at 37°C in darkness, and the reduction in absorbance at 517 nm, indicative of radical scavenging activity, was measured using a spectrophotometer. A standard curve was constructed using different concentrations of Trolox (ranging from 0.02 to 0.1 mM), and the results were expressed in mM Trolox equivalents (TE) per gram of dry weight sample corresponding to inhibition ratio of Trolox (%).

### 2.4 UPLC-trapped ion mobility-PASEF mass spectrometry

The LC separation was conducted on an ACQUITY UPLC HSS T3 column (2.1 × 100 mm, 1.8 μm, Waters, Milford, MA) using an ACQUITY UPLC™ system (Waters) with a flow rate of 0.3 mL min^-1^ at 40°C. The mobile phase comprised water containing 0.1% formic acid (solvent A) and acetonitrile (solvent B). The elution gradient program was as follows: 0–2 min, 10% B; 2–3 min, 10–15% B; 3–8 min, 15–20% B; 8–13.5 min, 20–30% B; 13.5–23 min, 30–50% B; 23–24.5 min 50–60% B; 24.5–28 min, 60–98% B. The mobile phase with 98% B was maintained for 4 min before returning to 10% B. The column was re-equilibrated with 10% B for 3 min before the next run. The UPLC system was coupled to a hybrid trapped ion mobility Q-TOF mass spectrometer (timsTOF fleX, Bruker Daltonics, Bremen, Germany). Ions were generated via a standard electrospray ionization (ESI) interface (Apollo II, Bruker Daltonics). The optimal MS settings included a capillary voltage of 4.5 kV for positive mode and –3.6 kV for negative mode, nebulizer pressure of 2.2 bar, drying gas flow rate of 10.0 L min^-1^, and dry temperature of 220°C. The drying gas also served as the concurrent trapped ion mobility spectrometry (TIMS) gas. Full-scan MS data were acquired in the mass range of 20–1300 Da with collision energy set between 20–50 eV in both positive and negative modes. The parallel accumulation–serial fragmentation (PASEF) method was employed to achieve maximum isolation efficiency and MS/MS speed of precursors at full sensitivity (Meier et al., 2015). For the TIMS analyzer, accumulation and ramp times were set to 100 ms each, and ion mobility was scanned from 0.45 to 1.45 Vs/cm^2^. One full TIMS-MS scan and two PASEF MS/MS scans were acquired within a cycle of 0.53 s. Before batch analyses of samples, the TIMS and MS analyzers were externally calibrated using a solution of 10 mM sodium formate and Agilent ESI-L Low Concentration Tune Mix (3:7 for positive mode, 7:3 for negative mode, v/v). The data were internally recalibrated with calibrants injected at the start of each sample analysis. The TIMS and MS operations were controlled and synchronized using the instrument control software timsControl ver. 4.5 (Bruker Daltonics).

### 2.5 Data processing for compound identification

The acquired PASEF MS/MS spectra were subjected to data processing for compound identification. Data extraction, recalibration and generation of bucket tables were conducted from the PASEF MS/MS files based on a feature finding algorithm (T-Rex 4D) in the MetaboScape ver. 6.0.2 (Bruker Daltonics), as previously described (Vasilopoulou et al., 2020). Feature detection was executed with the criteria of an intensity threshold of 300 counts and a minimum 4D peak size of 10 points when using recursive feature extraction. For ion deconvolution, typically EIC correlation was set to 0.8, and in positive ion mode, the primary ion ([M+H]^+^), seed ions ([M+Na]^+^ and [M+K]^+^) as well as the common ion ([M+H–H_2_O]^+^) were utilized. In negative ion mode, ion deconvolution was carried out using the primary ion ([M–H]^-^), seed ion ([M+Cl]^-^) and the common ion ([M–H–H_2_O]^-^). The tolerances for feature annotation in MetaboScape were as follows: m/z 5 ppm for narrow and 10 ppm for wide, mSigma 20 for narrow and 50 for wide, MS/MS 850 for narrow and 400 for wide. Based on these parameters the similarity between the spectral library and measured spectrum for annotated compounds of interest were evaluated. Spectral libraries including Bruker MetaboBASE^®^ Personal Library 3.0, Bruker HMDB Metabolite Library 2.0, Bruker NIST 2020 Mass Spectral Library, and Bruker Summer MetaboBASE Plant Library were employed for identification of phytochemicals. The classification of annotated compounds was done with the information of PubChem DB, and the subclass identification of statistically significant compounds were done using ClassyFire, an automated chemical classification tool (Djoumbou Feunang et al., 2016).

Multivariate statistical analysis was performed via the MetaboAnalyst 5.0 platform (https://www.metaboanalyst.ca/) (Xia et al., 2009). Variables in projection (VIP) scores, fold-changes, and *p*-values for each annotated compound were calculated using principal component analysis (PCA) and partial least squares-discriminant analysis (PLS-DA). By filtering important features based on VIP scores of 1 or more and a fold change greater than 2 or smaller than 0.5, significant compounds were from both ionization modes (positive 165, negative 75). To visually represent the selected significant features, heatmaps were generated. These heatmaps were categorized into distinct groups: Dokbulwang-dominant groups (G1 and G3) and Korean local cultivars-dominant groups (G2 and G4).

### 2.6 Metabolic network mapping analysis

Pair-wise Tanimoto chemical similarity coefficients were utilized to construct molecular networks using the MetaMapp platform (http://metamapp.fiehnlab.ucdavis.edu), following established procedures (Barupal et al., 2012). In brief, chemical similarity mapping was performed utilizing information on statistically significant compounds identified from the red chili pepper samples in this study, including PubChem ID, SMILES code, and compound name. The MetaMapp platform integrated chemical and biochemical similarity matrix files to establish connections among metabolites and cluster them within the network. The resulting network was visualized using the ‘organic layout’ algorithm implemented in Cytoscape 3.10 software (Shannon et al., 2003). Statistical thresholds (*p*-values) and fold-changes of the compounds were incorporated to emphasize the metabolic differences between various cultivars.

## 3 Results and discussion

### 3.1 Total flavonoid, phenolic, and antioxidant activities

Within the realm of plant phytochemicals, phenolic compounds hold diverse physiological functions, encompassing free radical scavenging, antibacterial, anticancer, and antioxidant activities (Mueen et al., 2010; Barad et al., 2014; Acidri et al., 2020; Qadi et al., 2020; Stuper-Szablewska et al., 2022; Bini et al., 2023). Flavonoids, featuring flavones as their fundamental structure, are present in flowers, stems, and fruits of plants and are recognized for their antioxidant, anticancer, and anti-inflammatory properties (Grassi et al., 2010; Yonekura-Sakakibara et al., 2019; Wen et al., 2020). Hence, the total flavonoid and phenolic contents (TFC and TPC) serve as potential indicators of specific physiological effects associated with natural products derived from plants. To compare the phytochemical contents of the local cultivars (Subicho and Eumseong), conventional estimation methods for TFC and TPC were applied in this study, as demonstrated in the Section 2.2 (Chandra et al., 2014). Additionally, antioxidant activity assays such as the oxygen radical absorption capacity (ORAC), the hydroxyl radical antioxidant capacity (HORAC), the total peroxyl radical trapping antioxidant parameter (TRAP), and the total oxyradical scavenging capacity (TOSC) tests have been widely utilized to assess antioxidant quality (Munteanu and Apetrei, 2021). Among these assays, the DPPH method is a simple, rapid and convenient method for screening of organic radical scavenging activity (Marinova and Batchvarov, 2011); therefore, it was employed in this study to evaluate the antioxidant activities of the corresponding phytochemicals.

**Figure 1:**
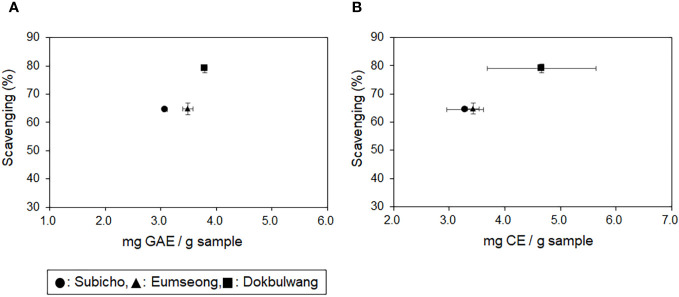
Total phenolic and flavonoid contents of Korean local red chili cultivars (Subicho and Eumseong) and Dokbulwang cultivar, along with their corresponding DPPH scavenging effects. **(A)** TPC versus scavenging efficiency and **(B)** TFC versus scavenging efficiency.

As indicated in the results of the TPC assay (refer to **Table 1**), the public cultivar exhibited the highest TPC levels (3.79 ± 0.04 mg GAE/g sample), followed by Eumseong (3.48 ± 0.09 mg GAE/g sample) and Subicho (3.07 ± 0.04 mg GAE/g sample). Additionally, the public cultivar displayed superior TFC levels (4.66 mg ± 0.98 CE/g sample) compared to the others (3.43 ± 0.11 mg CE/g sample in Eumseong, 3.28 ± 0.33 mg CE/g sample in Subicho), as detailed in **Table 2**. The radical scavenging efficiency assessed through the DPPH assay for the three different cultivars was as follows: Dokbulwang (79.05 ± 1.47%), Subicho (64.54 ± 0.56%), and Eumseong (64.85 ± 2.04%). These findings are illustrated in **Figure 1**, depicting the relationship between TFC, TPC, and scavenging efficiency among the cultivars.

**Table 1:** Anti-oxidant scavenging effects of three red chili peppers based on total phenolic contents.

Class	mg GAE/g sample	Scavenging efficiency (%)	Scavenging rate normalized by TPC
Dokbulwang	3.79 ± 0.04	79.05 ± 1.47	20.87
Subicho	3.07 ± 0.04	64.54 ± 0.56	21.02
Eumseong	3.48 ± 0.09	64.85 ± 2.04	18.61

**Table 2:** Anti-oxidant scavenging effects of three red chili peppers based on total flavonoid contents.

Class	mg CE/g sample	Scavenging efficiency (%)	Scavenging rate normalized by TFC
Dokbulwang	4.66 ± 0.98	79.05 ± 1.47	16.97
Subicho	3.28 ± 0.33	64.54 ± 0.56	19.67
Eumseong	3.43 ± 0.11	64.85 ± 2.04	18.90

To assess the scavenging efficiency of ethanolic extracts from different cultivars, the scavenging rates were determined by normalizing with their corresponding TPC or TFC values. When normalized by TPC, the scavenging rate of the public cultivar and the Subicho cultivar were similar (20.87 and 21.02, respectively), while Eumseong exhibited a slightly lower value (18.61). Interestingly, when normalized by TFC, the scavenging rate of Subicho and Eumseong cultivars was higher (19.67 and 18.90, respectively) compared to the public cultivar (16.97). These findings suggest that specific flavonoid components in the local cultivars possess stronger antioxidant potential. To gain further insights into the phytochemical composition of red chili peppers, high-resolution ion-mobility Q-TOF mass spectrometry (MS) analysis was conducted.

### 3.2 High-resolution ion-mobility Q-TOF MS profiling of ethanolic extracts

To comprehensively characterize the phytochemical profiles of two indigenous Korean local cultivars and compare them with a public cultivar, the 80% ethanolic extracts obtained from red pepper powder samples were analyzed using hybrid ion-mobility Q-TOF MS. The separation was initially performed via a UPLC system based on the constituents’ polarity through reversed-phase chromatography (see **Supplementary Figure S1**). Subsequently, the compounds were further separated based on their conformational differences in ion mobility spectroscopy, utilizing their collision cross section (CCS) values. This approach led to enhanced identification of complex compounds, as illustrated by the TIMS MS heat maps in **Supplementary Figure S2**. Similar methodologies have been successfully applied in previous studies, such as proteomics for peptides and metabolomics for metabolites (Meier et al., 2015; Schroeder et al., 2019; Vasilopoulou et al., 2020).

In the hybrid ion-mobility Q-TOF MS analysis, a total of 6779 and 3401 molecular features were detected from three red chili peppers in positive and negative ion modes, respectively. Among these, 458 compounds (6.8% of the total in positive mode) and 192 compounds (5.6% of the total in negative mode) were successfully identified by matching with a reference spectral library. These identified compounds were categorized into molecular classes, including amino acids, carbohydrates, lipids, organic compounds, phenolics, terpenoids, and others, as illustrated in **Supplementary Figures S3**–**S5**. Organic compounds and lipids appeared to be the most abundant molecular classes in all samples, followed by phenolics and/or terpenoids. Furthermore, a total of 621 compounds were detected across the three red chili pepper samples in both positive and negative ion modes. This diverse array of compounds encompassed 211 organic compounds (34.0% of the total), 204 lipids (32.9% of the total), 65 phenolics (10.5% of the total), 48 terpenoids (7.7% of the total), 26 amino acids (4.2% of the total), 23 carbohydrates (3.7% of the total), and 44 other compounds (7.1% of the total). Notably, 94.5% of the total compounds (587 compounds) were commonly observed in all cultivars, as depicted in **Supplementary Figure S6**. Specifically, cultivar-specific components (compounds exclusively found in each cultivar) constituted less than 1% of the total, indicating a qualitative similarity in phytochemical components across the cultivars. Further details regarding the chemical attributes of these compounds including RT, CCS and mass errors, can be found in the **Supplementary Datasets 1, 2**.

### 3.3 Comparison of phytochemical profiles

**Figure 2:**
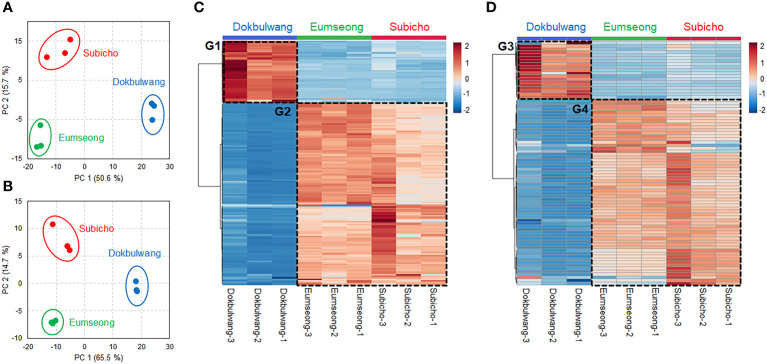
Principal component analysis (PCA) plots of phytochemicals identified from the local cultivars and the public cultivar. PCA plots with datasets by positive **(A)** and negative **(B)** ion modes. Heatmaps showed the local cultivars and the public cultivar-dominant compounds in positive **(C)** and negative **(D)** ion modes. The heatmaps were displayed with the compounds fitted in the criterion of *p*-value < 0.05, variable importance in projection (VIP) score > 1 and |fold change| > 2.

Despite the somewhat similar overall distribution of molecular classes of phytochemicals in Subicho and Eumseong cultivars compared to the public cultivar, principal component analysis (PCA) revealed distinct separation between the cultivars (refer to **Figures 2A, B**). Each cultivar exhibited clear separation, irrespective of the ion mode, and the local cultivars and the public cultivar were distinctly segregated by PC1 ordinations. To identify the key phytochemical constituents dominant in local cultivars, statistically significant compounds were selected and presented in heat maps. As depicted in **Figures 2C, D**, we identified local cultivar-dominant groups (G2 and G4) and public cultivar-dominant groups (G1 and G3). Further investigation into their molecular class distributions was conducted.

**Figure 3:**
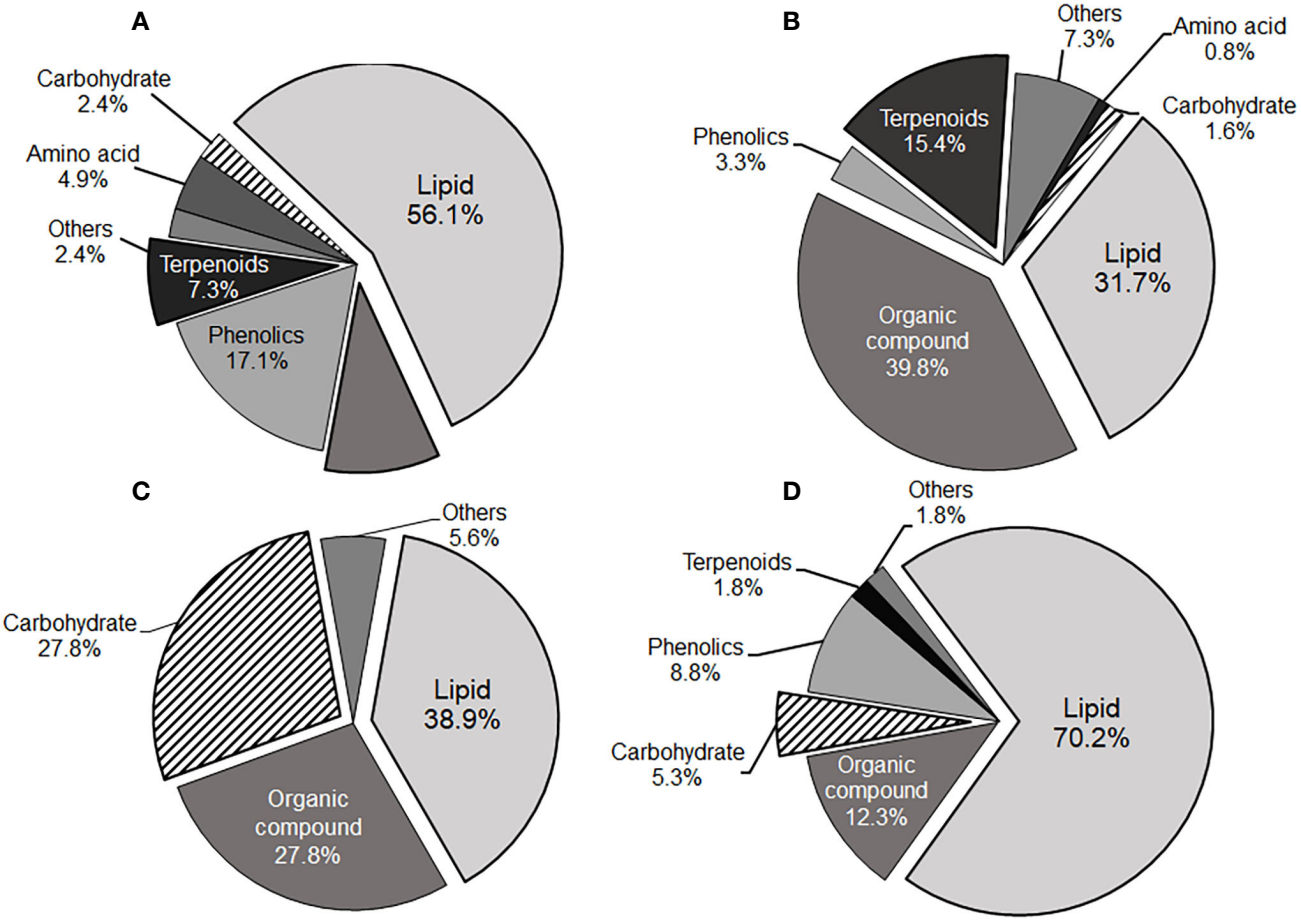
Pie charts illustrating the compound class distributions of the local cultivars-dominant **(B, D)** and the public cultivar-dominant **(A, C)** phytochemicals identified via positive and negative ion modes. **(A, B)** were derived from positive ion mode detection, whereas **(C, D)** were from negative ion mode detection.

In our study, lipid class compounds constituted the majority of phytochemicals in both the local cultivars (31.7% and 70.2% in positive and negative detections, respectively; see **Figures 3B, D**) and the public cultivar (56.1% and 38.9% in positive and negative detections, respectively; see **Figures 3A, C**). Notably, their chemical features were distinct. Specifically, carbaximidic acids (such as stearamide), fatty amides (such as palmitic amide), and linoleic acids were significantly observed in the local cultivars in both positive and negative detections. In contrast, the most abundant lipid species found in the public cultivars were glycerophosphocholines and glycerophosphoethanolamines in positive detection, and monoacylglycerophosphates and lysophosphatidylethanolamines in negative detection. Fatty acid composition is pivotal for the nutrient quality of peppers, with high levels of polyunsaturated fatty acids (PUFA) generally considered beneficial for health (Zaki et al., 2013; Sora et al., 2015). Consequently, the elevated levels of linoleic acids and its derivatives in the local cultivars could have significant implications for human health. Additionally, fatty acids and their amide derivatives have been recognized as inherent self-defense mechanisms among endophytes in natural environments (Tanvir et al., 2018).

Terpenoids, also known as isoprenoids, are isoprene-based natural products with diverse functions in the metabolism of various organisms, ranging from plants to animals (Bergman et al., 2019). Monoterpenoids and sesquiterpenoids play a vital role in plants’ defense mechanisms against external pests and pathogens, aiding in their protection. These compounds serve as defense mechanisms by either absorbing harmful substances from the air, physically deterring insects, or inhibiting the growth of pests and pathogens (Yu and Utsumi, 2009). Moreover, plants produce these compounds to adapt to environmental changes, aiding them in acclimating to various conditions such as high temperatures, dryness, and high altitudes (Martin et al., 2003; Morshedloo et al., 2017). Terpenoid compounds were notably found in the local cultivars, constituting 15.4% in positive detection (refer to **Figure 3B**). Specifically, sesquiterpenoids and diterpenoids compounds such as xanthorrhizol and abietic acid, were significantly observed in the local cultivars (see **Figure 3** and **Supplementary Tables S2**, **S3**). The abundance and diversity of terpenoids compounds in Korean local cultivars may be linked to enhanced plant tolerance and resistance to challenging environmental conditions (Choi et al., 2005; Costa et al., 2016).

The public cultivar also exhibited elevated levels of carbohydrates (27.8% in negative detection), including sucrose and galactinol (**Figure 3C**; **Supplementary Table S4**). Sucrose and galactinol are involved in the synthesis of raffinose family oligosaccharides in plants, and raffinose series oligosaccharides, expressed under non-biological stresses such as drought, play a role in germ protection (Sengupta et al., 2015). Traumatic acid, an endogenous molecule in plants, was notably abundant in the local cultivars within the G4 group. It has been identified as a wound hormone, stimulating cell division near wounded sites to form a protective callus and inducing the healing of damaged tissue (English et al., 1939).

**Figure 4:**
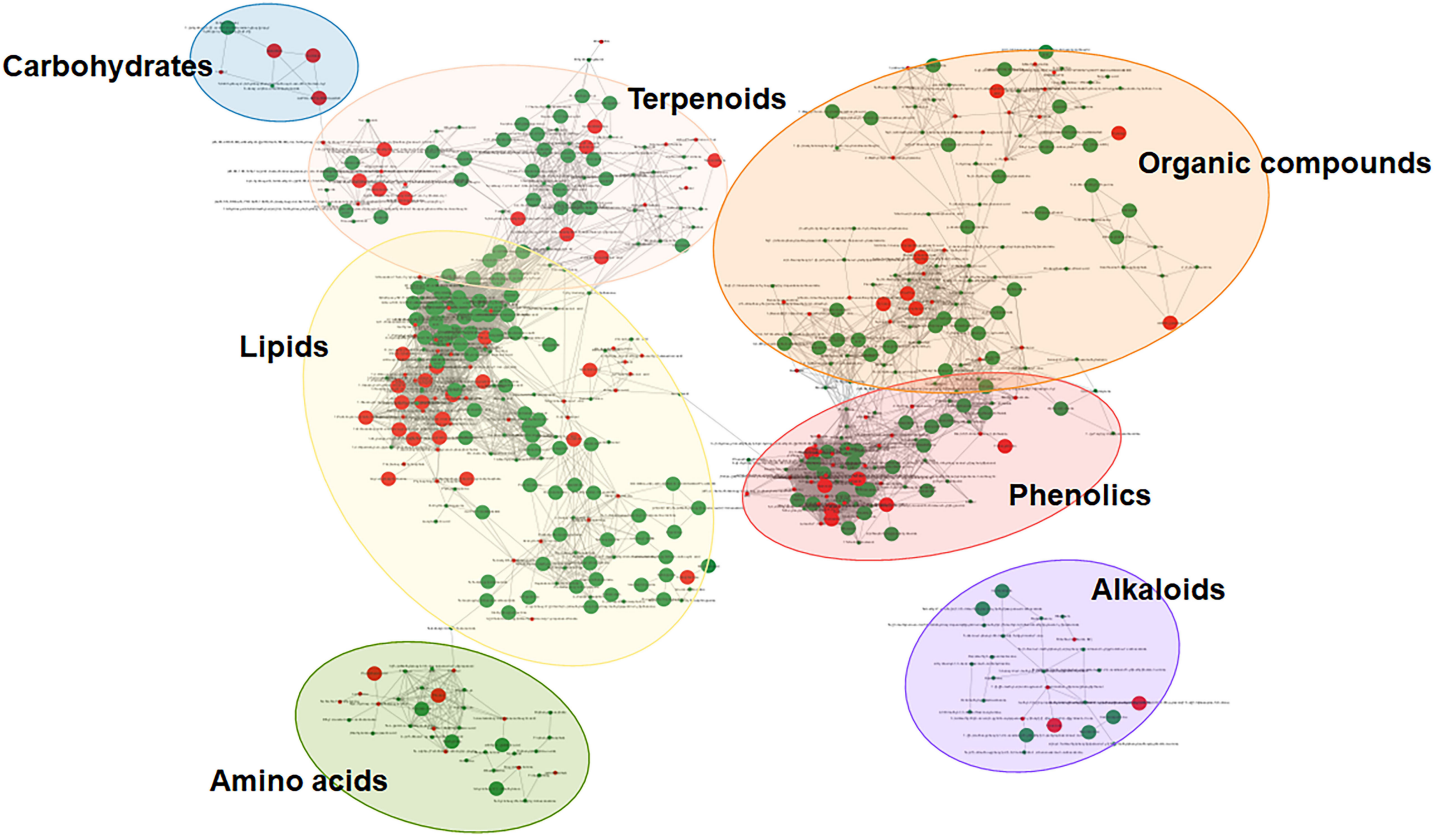
Molecular networks highlighting each cultivar-dominant phytochemicals in representative metabolic pathways. Green nodes represent more abundant phytochemicals in Korean local cultivars, and red nodes represent more abundant phytochemicals in public cultivar.

As demonstrated by **Figure 4**, the phytochemicals identified from three red chili pepper cultivars were scattered into seven different clusters of nodes using the combination of chemical similarity and biochemical reactant pair mapping. Mapping phytochemical data is highlighted for both local and public cultivars, displaying only significantly different phytochemical compounds (VIP > 1, |fold change| > 2, and *p* < 0.001) while not presenting the undifferentiated compounds. For clarity, phytochemical compounds of unknown structure have been excluded. The 148 annotated phytochemicals were clustered into seven major network clusters: carbohydrates, terpenoids, organic compounds, phenolics, alkaloids, amino acids, and lipids. This approach allowed for a comprehensive visualization and analysis of the metabolomic data, shedding light on the unique characteristics of the studied compounds within the different sample groups.

**Figure 5:**
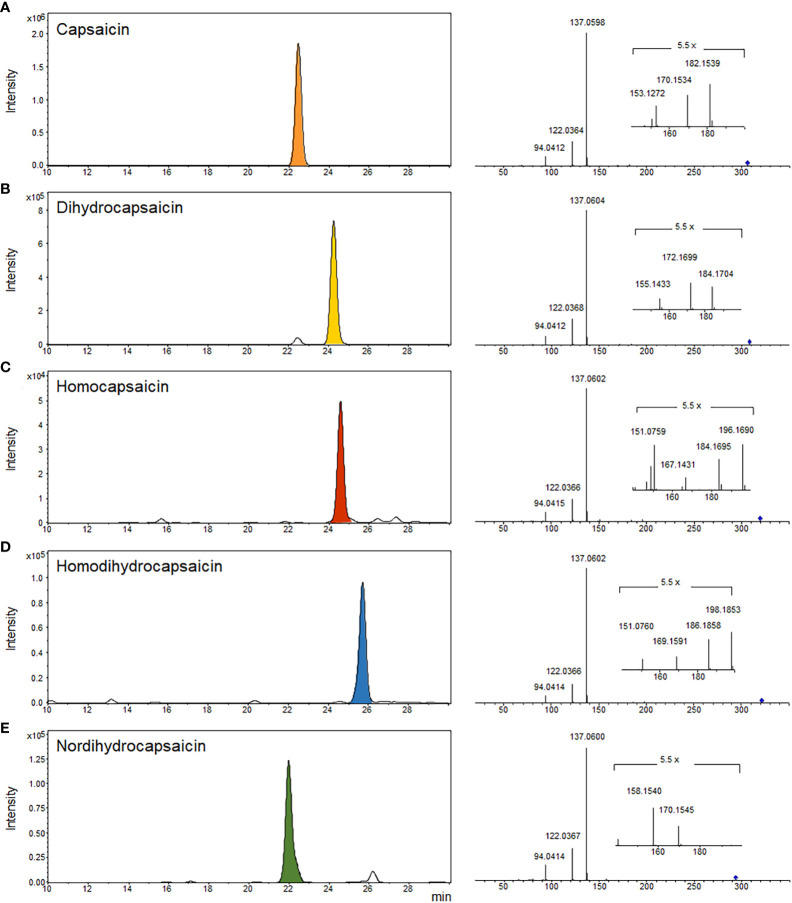
EIC and MS2 spectra of five major capsaicinoids in three red chili pepper cultivars. **(A)** capsaicin, **(B)** dihydrocapsaicin, **(C)** homocapsaicin, **(D)** homodihydrocapsaicin, and **(E)** nordihydrocapsaicin.

**Figure 6:**
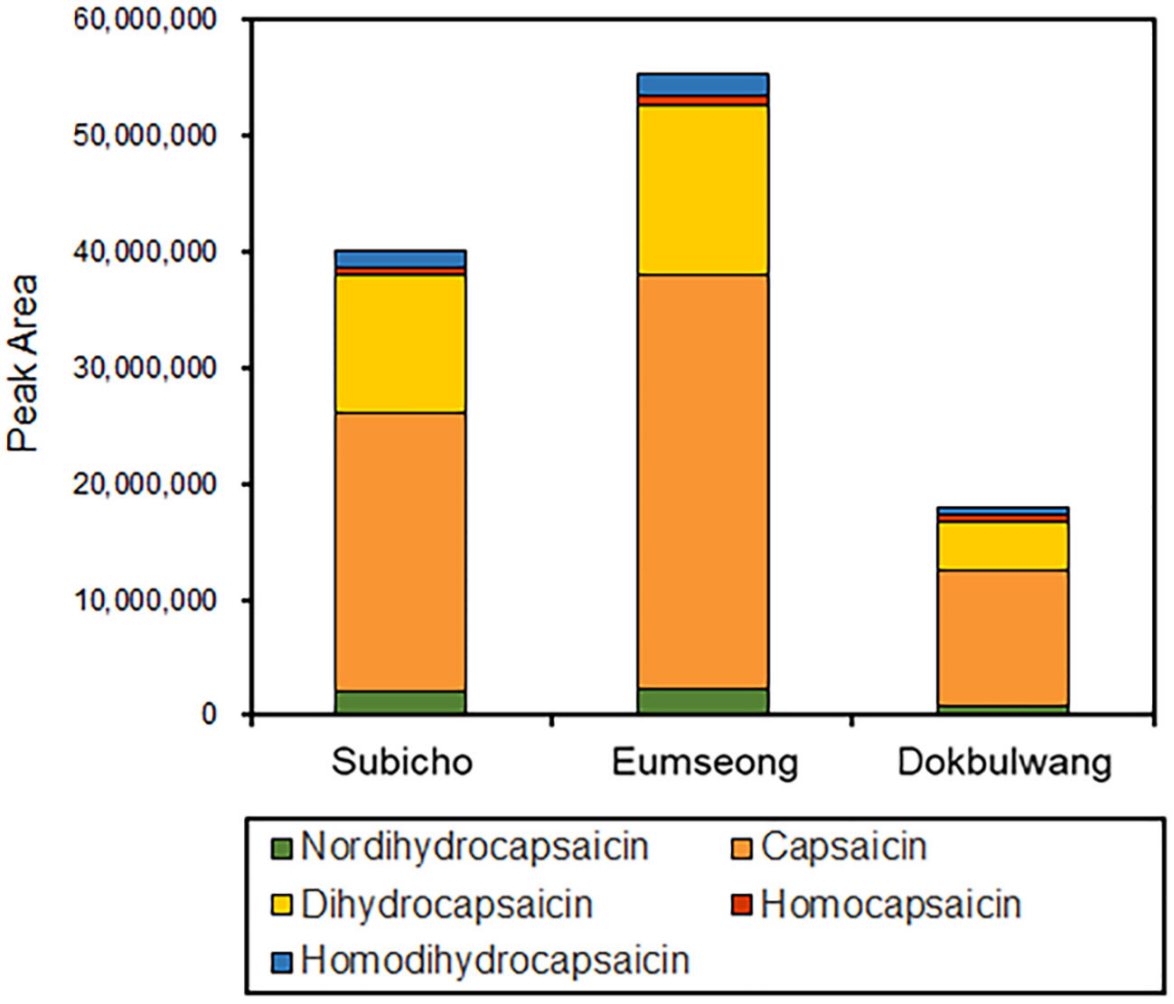
The sum of peak area of five major capsaicinoids in three red chili pepper cultivars.

In red chili pepper, capsaicins are noteworthy active ingredients, renowned for their antioxidant properties and pungent function. Specifically, capsaicin and dihydrocapsaicin constitute approximately 80-90% of the total capsaicinoid content in red chili pepper, with the remaining portion comprising nordihydrocapsaicin, homodihydrocapsaicin, and homocapsaicin (Schulze and Spiteller, 2009; Lu et al., 2020). In our study, we identified five major capsaicinoid compounds—capsaicin, dihydrocapsaicin, homocapsaicin, nordihydrocapsaicin, and homodihydrocapsaicin—from LC-MS/MS datasets (see **Figure 5**). These capsaicinoids were identified based on their parent ions and characteristic fragment ions. Common fragment ions at m/z 137, 122, and 94 correspond to the vanillyl moiety and its methylene dissociated form, consistent with previous reports (Schweiggert et al., 2006). The intensity-based proportions of these five major capsaicinoids in the total phytochemicals from Subicho, Eumseong, and Dokbulwang were 9.5%, 10.0%, and 6.2%, respectively (refer to **Figure 6**). Remarkably, based on the peak intensity of these capsaicinoids, the local cultivars exhibited two to three times higher levels than the public cultivar. Consequently, the local cultivars possess significantly higher contents of capsaicinoid compounds, the active ingredients responsible for red chili pepper’s pungent flavor, compared to the public cultivar. These elevated levels of capsaicinoids in the local cultivars contribute significantly to their unique pungent flavors.

## 4 Conclusion

This study provides a comprehensive analysis of the phytochemical profiles of two Korean local cultivars of red chili pepper (Subicho and Eumseong, referred to as the local cultivars) in comparison to a typical phytophthora disease resistance cultivar (Dokbulwang, the public cultivar). Utilizing a hybrid ion-mobility Q-TOF MS analysis, an in-depth characterization of the complex phytochemical profiles present in the ethanolic extracts of these distinct cultivars were conducted.

Compared to the public cultivar, the local cultivars were identified as rich sources of sesquiterpenoids and diterpenoids. Terpenoids, known for their association with plant tolerance and resistance to pests and pathogens, have the potential for further development as biological pest control agents (Boncan et al., 2020; Ngegba et al., 2022). Linoleic acid, specifically found in local cultivars, can also find applications in industries such as cosmetics and pharmaceuticals (Chouaibi et al., 2019). Notably, the local cultivars exhibited higher levels of major capsaicinoid components (i.e., capsaicin, dihydrocapsaicin, homocapsaicin, nordihydrocapsaicin, and homodihydrocapsaicin) compared to common varieties. The elevated content of major capsaicinoids in local cultivars may be associated with their distinctive spiciness, and further investigations into the intricate relationships between capsaicinoid structures and their defensive functions will not only contribute to a broader understanding of plant defense mechanisms but may also have implications for agriculture and pest management strategies.

However, even though the hybrid ion-mobility Q-TOF MS provides higher resolution to effectively resolve molecular features within complex mixtures, the identification of phytochemical compounds in red pepper cultivars was impeded due to the lack and inaccuracy of reference spectral libraries. To address these challenges, several strategies, such as the creation of specialized databases and utilization of diverse matching algorithms, were implemented to enhance annotation coverage (Sawada et al., 2012; Bittremieux et al., 2022). In addition, the development of an in-house phytochemical spectral library using commercially available phytochemical standards is further planned. Furthermore, leveraging the 4-dimensional attributes, namely MS1, MS2, retention time (RT), and collision cross section (CCS), of phytochemicals facilitated more precise annotation of compounds of interest within natural product extracts. Consequently, the implementation of these methods will lead to the identification of additional compounds, thereby overcoming the limitations posed by the existing spectral library.

The public cultivar showed higher TPC and TFC values than the local cultivars as well as the DPPH scavenging activity, but the TPC or TFC-normalized values of the anti-oxidant activity was higher for the local cultivars. Although the total contents of phenolic and flavonoid of local cultivars were lower than those of the public, the antioxidant efficiency of the local cultivars (especially for Subicho) were higher than the public cultivar, supporting the presence of stronger antioxidant compounds in the local cultivars. Furthermore, the unique chemodiversity observed within the pepper cultivars would allow us to deeply understand their physiological characteristics.

Based on these findings, the Korean local cultivars of red chili pepper appear to possess distinct properties compared to the typical phytophthora disease resistance cultivar. Further exploration of the bioactive compounds in these local cultivars holds significant potential for the advancement of native crops. These results not only enhance our comprehension of the health benefits and pungent properties of red chili pepper but also offer valuable insights for the development of functional foods and dietary supplements within the food industry.

## Data availability statement

The original contributions presented in the study are included in the article/**Supplementary Materials**, further inquiries can be directed to the corresponding author.

## Author contributions

HJ: Conceptualization, Methodology, Visualization, Writing – original draft. MC: Methodology, Data curation, Writing – review & editing. KJ: Resources, Supervision, Writing – review & editing.
