# Supplementary material for: Comprehensive phytochemical profiles and antioxidant activity of Korean local cultivars of red chili pepper (Capsicum annuum L.)
Source: Front Plant Sci. 2024 Jan 22;15:1333035. doi: 10.3389/fpls.2024.1333035 (PMC10840139; doi:10.3389/fpls.2024.1333035)
Supplement: Supplementary file 1 [file DataSheet_1.pdf]

Supplementary Material

Comprehensive phytochemical profiles and antioxidant activity of Korean local cultivars of red chili pepper (*Capsicum annuum L.*)

Hyemi Jang[1,2], Mira Choi[1*], Kyoung-Soon Jang[1,2*]

[1]Bio-Chemical Analysis Team, Korea Basic Science Institute, Cheongju, Republic of Korea

[2]Division of Bio-Analytical Science, University of Science and Technology, Daejeon, Republic of Korea

*** Correspondence:** Kyoung-Soon Jang, Ph.D.: ksjang@kbsi.re.kr

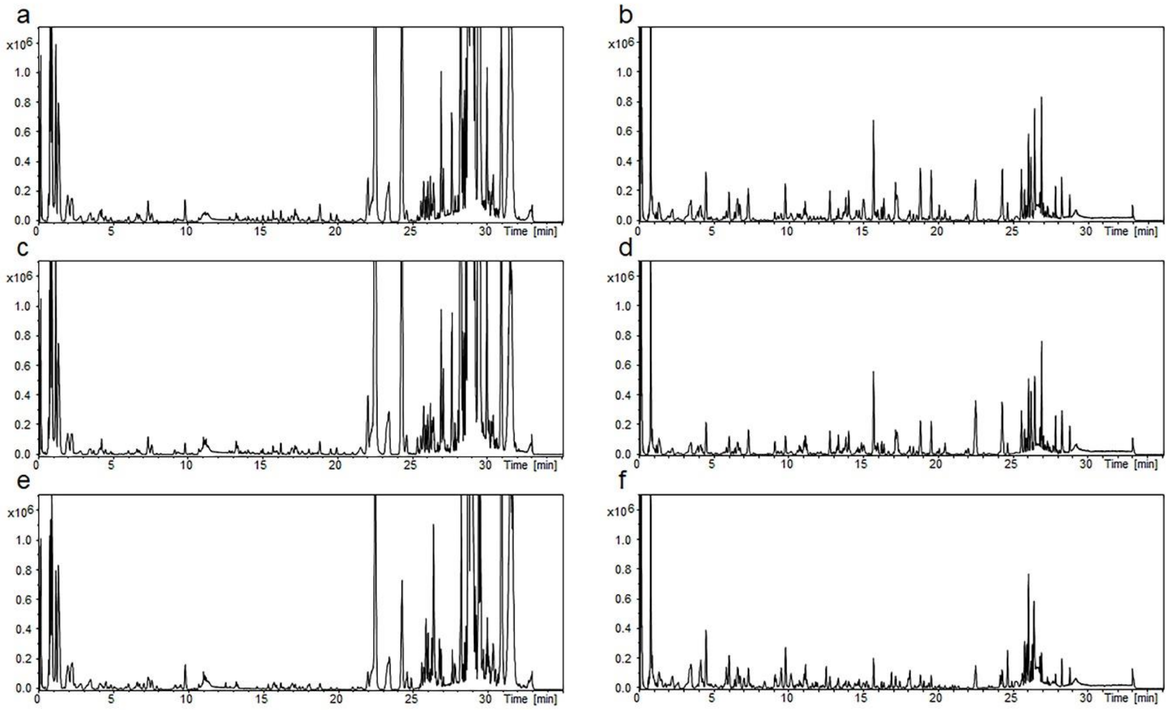

Supplementary Figure S1. Total ion chromatograms obtained from Korean local cultivars of red chili pepper (Subicho and Eumseong) and Dokbulwang cultivar via positive (a, c, and e) and negative (b, d, and f) ion modes. a and b, Subicho; c and d, Eumseong; e and f, Dokbulwang.

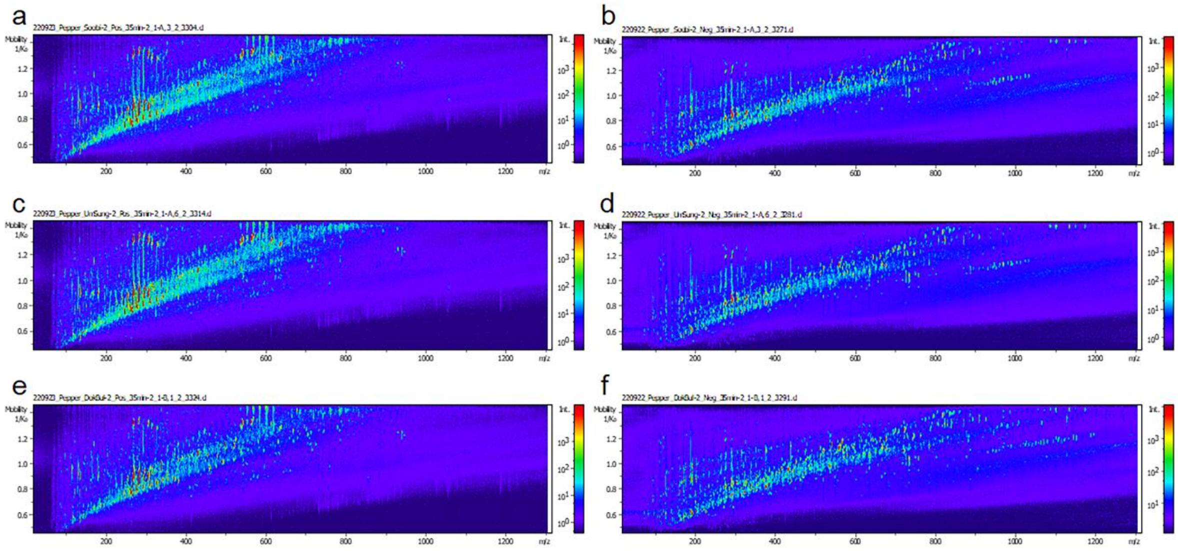

Supplementary Figure S2. TIMS MS Heat maps plotting m/z and $1/k_0$ of molecular features observed via positive (a, c, and e) and negative (b, d, and f) ion modes from Korean local cultivars of red chili pepper (Subicho and Eumseong) and Dokbulwang cultivar. a and b, Subicho; c and d, Eumseong; e and f, Dokbulwang.

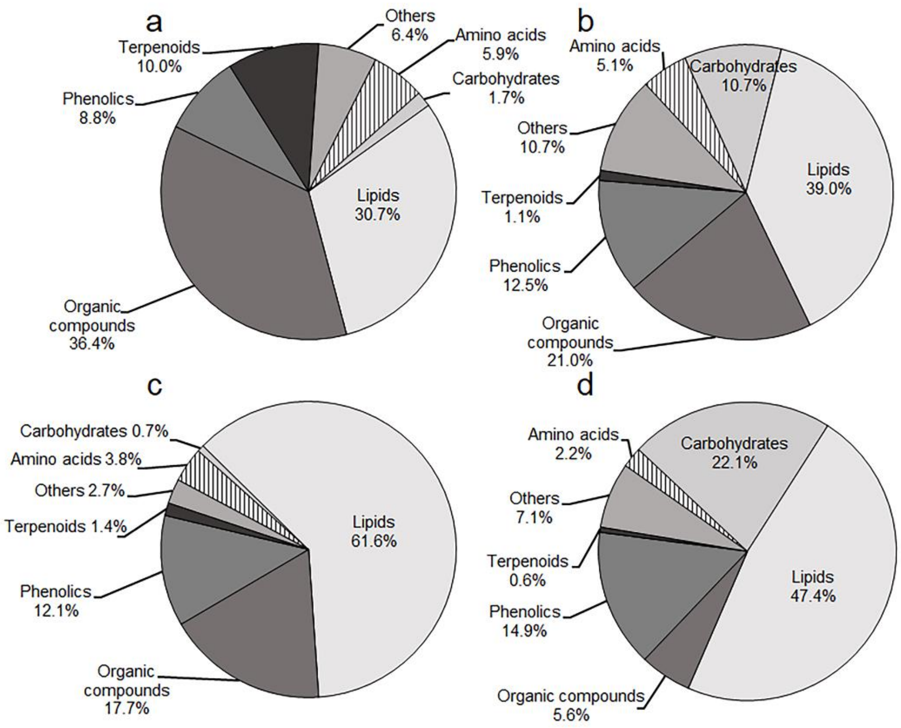

Supplementary Figure S3. Pie charts showing the distribution of molecular classes of phytochemical compounds identified by positive (a, c) and negative (b, d) ion modes from Subicho cultivar. The contribution of each of these groups was estimated based on the LC-MS peak-frequency (a and b) or peak-intensity (c and d)-weighted percentage of the total.

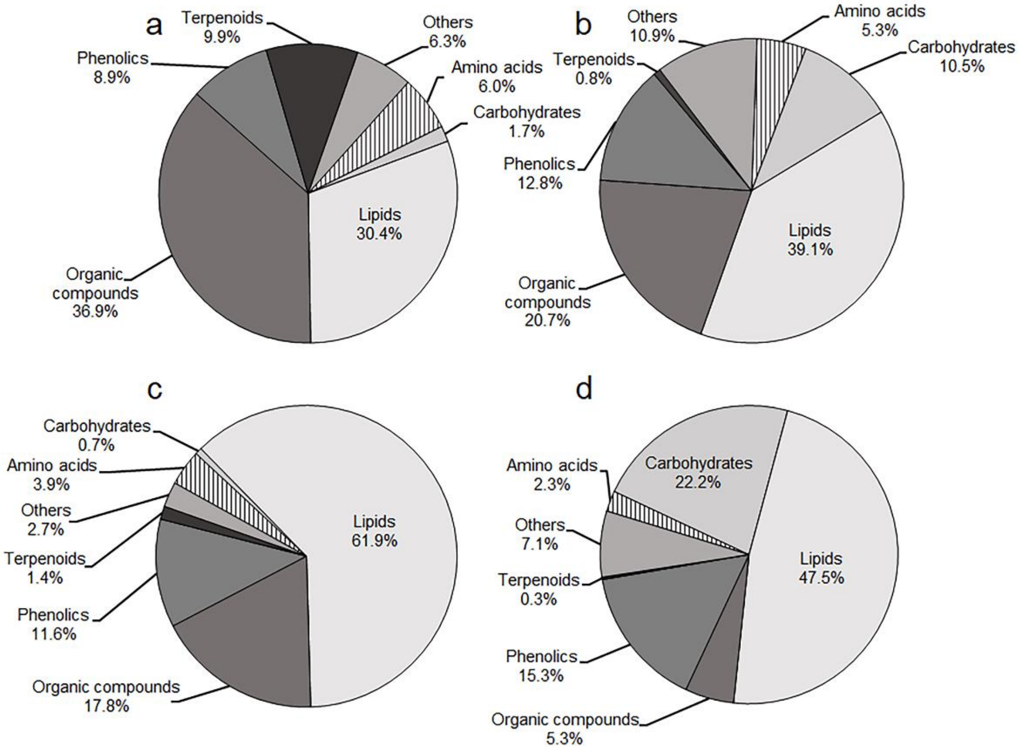

Supplementary Figure S4. Pie charts showing the distribution of molecular classes of phytochemical compounds identified by positive (a, c) and negative (b, d) ion modes from Eumseong cultivar. The contribution of each of these groups was estimated based on the LC-MS peak-frequency (a and b) or peak-intensity (c and d)-weighted percentage of the total.

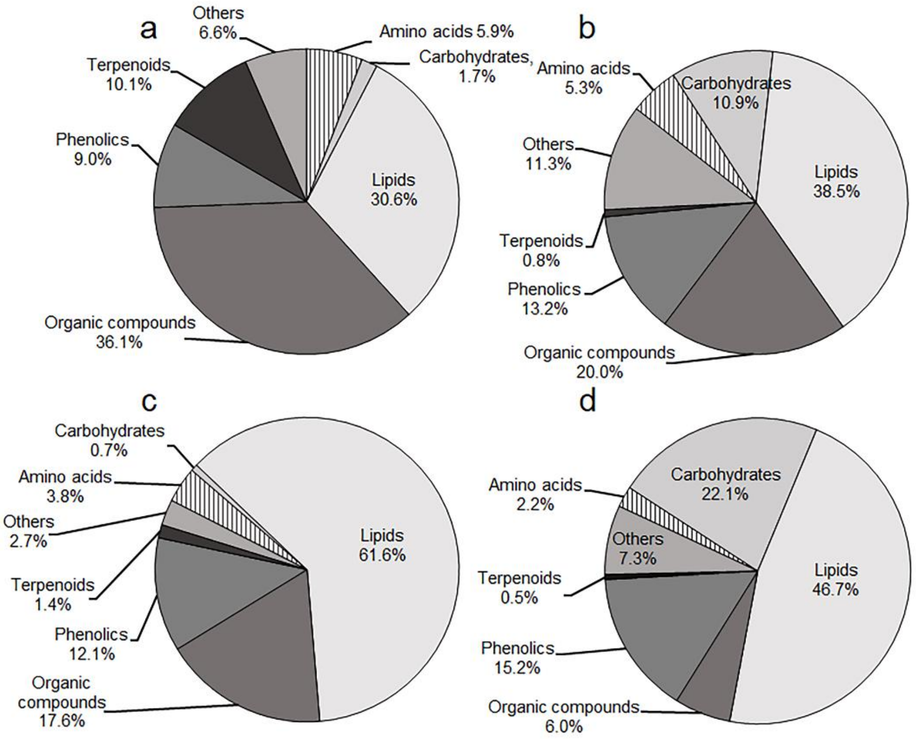

Supplementary Figure S5. Pie charts showing the distribution of molecular classes of phytochemical compounds identified by positive (a, c) and negative (b, d) ion modes from Dokbulwang cultivar. The contribution of each of these groups was estimated based on the LC-MS peak-frequency (a and b) or peak-intensity (c and d)-weighted percentage of the total.

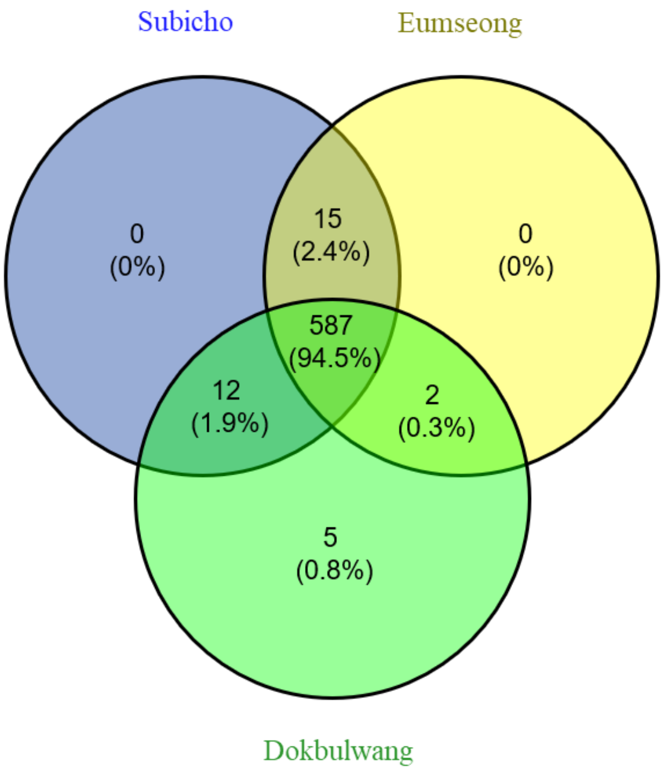

Supplementary Figure S6. Venn diagrams displaying the number of phytochemical compounds identified from three red chili pepper cultivars. Korean local cultivars (Subicho and Eumseong) and a common phytophthora disease resistance cultivar (Dokbulwang).

Supplementary Table S1. Compound class distributions of the cultivar-dominant phytochemicals. G1 and G3 denote the Dokbulwang cultivar-dominant compound classes, while G2 and G4 indicate the local cultivars-dominant compound classes.

Group \ Class		Amino acids	Carbohydrates	Lipids	Organic compounds	Phenolics	Terpenoids	Others
Positive mode	G1 – Public cultivar	2 (4.8%)	1 (2.4%)	24 (56.2%)	4 (9.6%)	7 (16.7%)	3 (7.2%)	1 (2.4%)
	G2 – Local cultivar	1 (0.9%)	2 (1.7%)	39 (32.8%)	48 (40.4%)	3 (2.6%)	18 (15.2%)	8 (6.8%)
Negative mode	G3 – Public cultivar	-	5 (27.8%)	7 (38.9%)	5 (27.8%)	-	-	1 (5.6%)
	G4 – Local cultivar	-	3 (5.3%)	40 (70.2%)	7 (12.3%)	5 (8.8%)	1 (1.8%)	1 (1.8%)
